# Entropy Measures for Stochastic Processes with Applications in Functional Anomaly Detection

**DOI:** 10.3390/e20010033

**Published:** 2018-01-11

**Authors:** Gabriel Martos, Nicolás Hernández, Alberto Muñoz, Javier M. Moguerza

**Affiliations:** 1 Facultad de Ciencias Exactas y Naturales, Universidad de Buenos Aires and CONICET, Buenos Aires C1428EGA, Argentina; 2 Department of Statistics, Universidad Carlos III de Madrid, 28903 Getafe, Spain; 3 Department of Computer Science and Statistics, University Rey Juan Carlos, 28933 Móstoles, Spain

**Keywords:** entropy, stochastic process, minimum-entropy sets, anomaly detection, functional data

## Abstract

We propose a definition of entropy for stochastic processes. We provide a reproducing kernel Hilbert space model to estimate entropy from a random sample of realizations of a stochastic process, namely functional data, and introduce two approaches to estimate minimum entropy sets. These sets are relevant to detect anomalous or outlier functional data. A numerical experiment illustrates the performance of the proposed method; in addition, we conduct an analysis of mortality rate curves as an interesting application in a real-data context to explore functional anomaly detection.

## 1. Introduction

The family of α-entropies, originally proposed by Rényi [1], plays an important role in information theory and statistics. Consider a random variable *Z* distributed according to a measure *F* that admits a probability density function *f*. Then, for α≥0 and α≠1, the α-entropy of *Z* is computed as follows:

(1) $$H_{\alpha }\left(Z\right)=\frac{1}{1-\alpha }\log\left(V_{\alpha }\left(Z\right)\right)$$

where $V_{\alpha }\left(Z\right)=E_{F}\left\{f^{\alpha -1}\right\}$, and $E_{F}$ stands for the expected value with respect to the *F* measure. Several renowned entropy measures in the statistical literature are particular cases in the family of α-entropies. For instance, when α=0, we obtain the Hartley entropy; when α→1, then $H_{\alpha }$ converges to the Shannon entropy; and when α→∞, then $H_{\alpha }$ converges to the Min-entropy measure. The contribution of this paper is two-fold. Firstly, we propose a natural definition of entropy for stochastic processes that extends the previous one and a suitable sample estimator for the observation of partial realizations of the process, the typical framework when dealing with functional data. We also show that Minimal Entropy Sets (MES), as formally defined in Section 3, are useful to solve anomaly detection problems, a common task in almost all data analysis contexts.

The paper is structured as follows: In Section 2, we introduce a definition of entropy for a stochastic process and suitable sample estimators for this measure. In Section 3, we show how to estimate minimum-entropy sets of a stochastic process in order to discover atypical functional data in a sample. Section 4 illustrates the theory with simulations and examples, and Section 5 concludes the work.

## 2. Entropy of a Stochastic Process

In this section, we extend the definition of entropy to a stochastic process. For the sequel, let (Ω,F,P) be a probability space, where F is the σ-algebra in Ω and *P* a σ-finite measure. We consider random elements (functions) X(ω,t):Ω×T→R in a metric space (T,τ). As usual in the case of functional data, the realizations of the random elements X(ω,·) are assumed in C(T), the space of real continuous functions in a compact domain T⊂R endowed with the uniform metric.

The first step is to consider a suitable representation for the stochastic process. We make use of the well-known Karhunen–Loève expansion [2] (p. 25, Theorem 1.5). Let X(ω,t) be a centered (zero-mean) stochastic process with continuous covariance function $K_{X}\left(s,t\right)=E\left(X\left(\omega ,s\right)X\left(\omega ,t\right)\right)$, then there exists a basis ${\left\{e_{i}\right\}}_{i\geq 1}$ of C(T) such that for all t∈T:

(2) $$X\left(\omega ,t\right)=\sum_{i=1}^{\infty }\xi_{i}\left(\omega \right)e_{i}\left(t\right),$$

where the sequence of random coefficients $\xi_{i}\left(\omega \right)=\int_{T}X\left(\omega ,t\right)e_{i}\left(t\right)dt$ comprises zero mean random variables with (co)variance $E\left(\xi_{i}\xi_{j}\right)=\delta_{ij}\lambda_{j}$, being $\delta_{ij}$ the Kronecker delta and ${\left\{\lambda \right\}}_{j\geq 1}$ the sequence of eigenvalues associated with the eigenfunctions of $K_{X}\left(s,t\right)$.

The equality in Equation (2) must be understood in the mean square sense, that is:

(3) $$\lim_{d\to \infty }E\left\{{\left(X\left(\omega ,t\right)-\sum_{i=1}^{d}\xi_{i}\left(\omega \right)e_{i}\left(t\right)\right)}^{2}\right\}=0,$$

uniformly in *T*. Therefore, we can always consider a ε-near representation $X_{d}\left(\omega ,t\right)=\sum_{i=1}^{d}\xi_{i}\left(\omega \right)e_{i}\left(t\right)$ such that for all ε arbitrarily small, there exists an integer *D* such that for d≥D, then $\tau \left(X,X_{d}\right)=\sup_{t\in T}\left|X\left(\omega ,t\right)-X_{d}\left(\omega ,t\right)\right|\leq \varepsilon$. From this result, it is possible to establish a suitable way to approximate the entropy of a random element X(ω,t) according to the distribution of the “representation coefficients” ${\left\{\xi_{i}\left(\omega \right)\right\}}_{i}^{d}$ obtained from $X_{d}\left(\omega ,t\right)$.

**Definition** **1** (*d*-truncated entropy for stochastic processes)**.** *Let X be a centered stochastic process with a continuous covariance function. Consider the truncation $X_{d}\left(\omega ,t\right)=\sum_{i=1}^{d}\xi_{i}\left(\omega \right)e_{i}\left(t\right)$ and the random vector $Z=(\xi_{1},\ldots ,\xi_{d})$; then, the d-truncated entropy of X is defined as $H_{\alpha }\left(X,d\right)=H_{\alpha }\left(Z\right)$.*

The “approximation error” when computing the entropy of the stochastic process *X* with Definition 1 decreases monotonically with the number of terms retained in the Karhunen–Loève expansion, at a rate that depends on the decay of the spectrum of the covariance function $K_{X}\left(s,t\right)$. In general, the more autocorrelated the process is, the more quickly the eigenvalues of $K_{X}\left(s,t\right)$ converge to zero. In practical functional data applications (see for instance the mortality-rate curves in Section 4), the autocorrelation is usually strong, and the truncation parameter *d* will be small when approximating the entropy of the process. The next example illustrates the definition.

**Example** **1.** *[Gaussian process] When X is a Gaussian Process (GP), the coefficients in the Karhunen–Loève expansion have the further property that they are independent and zero-mean normally distributed random variables. Therefore, the Shannon entropy (α=1) of X can be approximated with the truncated version of the GP as follows:*

$$H_{1}\left(X,d\right)=\frac{1}{2}\log{\left(2\pi e\right)}^{d}\det\left(\Sigma \right),$$

*where* Σ *is the diagonal covariance matrix with elements ${\left[\Sigma \right]}_{i,j}=E\left(\xi_{i}\xi_{j}\right)$ for i,j=1,…,d.*

In practice, we can only observe some realizations of the stochastic process *X*, and these observations are sparsely registered. Therefore, to estimate the entropy of X(ω,t) from a random sample of discrete realizations of a stochastic process, a first task is the representation of these paths by means of continuous functions. To this end, we consider a reproducing kernel Hilbert space H of functions, associated with a positive definite and symmetric kernel function K:T×T→R.

### Estimating Entropy in a Reproducing Kernel Hilbert Space

Most functional data analysis approaches for representing raw data suggest proceeding as follows: (i) choose an orthogonal basis of functions $\Phi =\{\phi_{1},\ldots ,\phi_{N}\}$, where each $\phi_{i}$ belongs to a general function space H; and (ii) represent each functional datum by means of a linear combination in the Span(Φ) [3,4]. Our choice is to consider H as a Reproducing Kernel Hilbert Space (RKHS) of functions [5]. In this case, the elements in the spanning set Φ are the eigenfunctions associated with the positive-definite and symmetric kernel function K:T×T→R that span H [5] (Moore-Aronszajn Theorem p. 19).

In our setting, the functional representation problem can be framed as follows: We have available *m* discrete observations, that is a realization path $x\left(t_{1}\right),\ldots ,x\left(t_{m}\right)$ of the stochastic element X(ω,t). We also assume that the discrete path ${\left\{x\left(t_{i}\right),t_{i}\right\}}_{i=1}^{m}$, as usual when dealing with real data, contains zero mean iid error measurements. Then, the functional data estimator, denoted onwards as $\tilde{x}\left(t\right)$, is obtained solving the following regularization problem:

(4) $$\tilde{x}\left(t\right):=\arg\min_{g\in H}\sum_{i=1}^{m}V{\left(x\left(t_{i}\right),g\left(t_{i}\right)\right)}^{2}+\gamma \Omega \left(g\right),$$

where *V* is a strictly convex functional with respect to the second argument, γ>0 is a regularization parameter, frequently chosen by cross-validation, and Ω(g) is a regularization term. By the representer theorem [6,7] (Theorem 5.2, p. 91, Proposition 8, p. 51), the solution of the problem stated in Equation (4) exists, is unique and admits a representation of the form:

(5) $$\tilde{x}\left(t\right)=\sum_{i=1}^{m}a_{i}K\left(t,t_{i}\right).$$

In the particular case of a squared loss function $V\left(w,z\right)={\left(w-z\right)}^{2}$ and considering $\Omega \left(g\right)=\int_{T}g^{2}\left(t\right)\;dt$, the coefficients of the linear combination in Equation (5) are obtained solving the following system:

(6) (γmI+K)a=y,

where $a={\left(a_{1},\ldots ,a_{m}\right)}^{T}$, $y={\left(x\left(t_{1}\right),\ldots ,x\left(t_{m}\right)\right)}^{T}$, I is the identity matrix of order *m* and K is the Gram matrix with the kernel evaluations, ${\left[K\right]}_{k,l}=K\left(t_{k},t_{l}\right)$, for k=1,…,m and l=1,…,m. To relate the Karhunen–Loève expansion in Equation (2) to the RKHS representation, we make use of Mercer’s theorem [2] (Lemma 1.3, p. 24), then $K_{X}\left(s,t\right)=\sum_{j=1}^{\infty }\lambda_{j}\phi_{j}\left(s\right)\phi_{j}\left(t\right)$, where $\lambda_{j}$ is the eigenvalue associated with the orthonormal eigenfunction $\phi_{j}$ for j≥1, and invoking the reproducing property, then:

(7) $$\begin{array}{cc} X(\omega ,t) & =\langle X\left(\omega ,s\right),K_{X}\left(s,t\right)\rangle \\ & =\sum_{j=1}^{\infty }\lambda_{j}\phi_{j}\left(t\right)\int_{T}X\left(\omega ,s\right)\phi_{j}\left(s\right)ds. \end{array}$$

Therefore, following Equation (2), $\xi_{j}\left(\omega \right):=\sqrt{\lambda_{j}}\int_{T}X\left(\omega ,s\right)\phi_{j}\left(s\right)ds$ and $e_{j}\left(t\right)=\sqrt{\lambda_{j}}\phi_{j}\left(t\right)$; and the connection is clearly established. When working with discrete realizations of a stochastic process, we must solve two sequential tasks. First, we need to represent raw data as functional data and later find a truncated representation of the function. To this end, when combining Equation (5) with Mercer’s theorem and the reproducing property, we obtain:

$${\tilde{x}}_{d}\left(t\right)=\sum_{j=1}^{d}\sqrt{\lambda_{j}}\phi_{j}\left(t\right)\sqrt{\lambda_{j}}\left(\sum_{i=1}^{m}a_{i}\phi_{j}\left(t_{i}\right)\right),$$

and now, $z_{j}:=\sqrt{\lambda_{j}}\sum_{i=1}^{m}a_{i}\phi_{j}\left(t_{i}\right)$ is the realization of the random variable $\xi_{j}$ for j=1,…,d; see [8] for further details. For some kernel functions, for instance the Gaussian kernel, the associated sequence of eigen-pairs $\left(\lambda_{j},\phi_{j}\right)$ for j≥1 is known [9] (pp. 10), and we can obtain an explicit value for all $z_{j}$. If not, let $\left(\lambda_{j},v_{j}\right)$ be the *j*-eigenpair associated with the kernel matrix $K\in R^{m\times m}$, then $z_{j}=\sqrt{\lambda_{j}}\sum_{i=1}^{m}a_{i}v_{i,j}$ for j=1,…,d.

In practice, given a sample of *n* discrete paths (realizations) of the stochastic process *X*, say $\left\{x_{l}\left(t_{1}\right),\ldots ,x_{l}\left(t_{m}\right)\right\}$ for l=1,…,n, a suitable input to estimate entropy in Definition 1 is to consider the set of multivariate vectors $z_{l}=\left(z_{l,1},\ldots ,z_{l,d}\right)$ for l=1,…,n, as formally proposed in the next definition.

**Definition** **2** (*K*-entropy estimation of a stochastic process)**.** *Let $\left\{x_{1}\left(t_{i}\right),\ldots ,x_{n}\left(t_{i}\right)\right\}$ for i=1,…,m be a discrete random sample of X, and let ${\left\{\left(\lambda_{j},v_{j}\right)\right\}}_{j=1}^{d}$ be the eigen-pairs of the kernel matrix $K\in R^{m\times m}$, where d=rank(K). Consider the corresponding finite dimensional representation $S_{n}:=\left\{z_{1},\ldots ,z_{n}\right\}$, where $z_{l}=\left(z_{l,1},\ldots ,z_{l,d}\right)\in R^{d}$ for l=1,…,n and $z_{l,j}=\sqrt{\lambda_{l,j}}\sum_{i=1}^{m}a_{l,i}v_{i,j}$ for j=1,…,d. Then, the estimated kernel entropy of X is defined as ${\hat{H}}_{\alpha }\left(X,K\right)={\hat{H}}_{\alpha }\left(Z\right)$.*

In Definition 2, ${\hat{H}}_{\alpha }\left(Z\right)$ denotes the estimated entropy using the (finite dimensional) representation coefficients $S_{n}=\left\{z_{1},\ldots ,z_{n}\right\}$. In Section 3, we formally introduce two approaches to estimate entropy departing from $S_{n}$. The next example illustrates the estimation procedure in the context of GPs in Example 1.

**Illustration** **with** **Example** **1:** Consider 100 realizations of a GP as follows: 50 curves from $X\left(t\right)=\sum_{i=1}^{3}\xi_{i}e_{i}\left(t\right)$ and another 50 curves from $Y\left(t\right)=\sum_{i=1}^{3}\zeta_{i}e_{i}\left(t\right)$; where $e_{i}\left(t\right)$ is a Fourier basis in T=[0,1], $\xi_{i}∼N\left(\mu =0,\sigma^{2}=0.5\right)$, and $\zeta_{i}∼N\left(\mu =0,\sigma^{2}=2\right)$ are independent normally distributed random variables (r.v.) for i=1,2,3.

**Figure 1:**
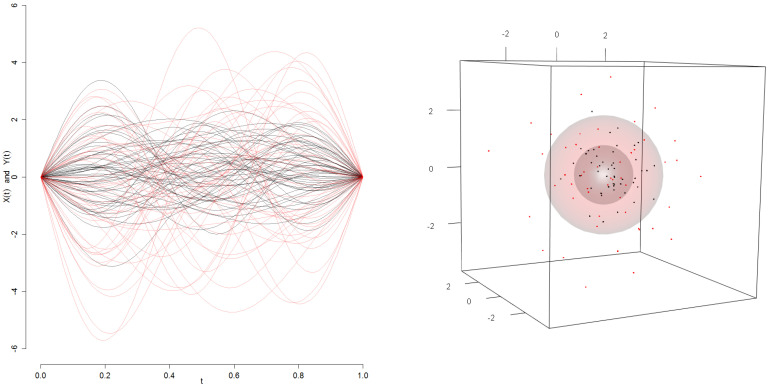
Gaussian processes realizations on the left and coefficients for entropy estimation on the right. The sizes of the balls on the right are proportional to the determinants of ${\hat{\Sigma }}_{X}$ (in black) and ${\hat{\Sigma }}_{Y}$ (in red).

In Figure 1 (left), we illustrate the realizations of the stochastic processes, in black (“—”) the sample paths of X(t) and in red (“—”) the paths corresponding to Y(t). In Figure 1 (right), we show the distribution of the linear combination coefficients ${\left\{{\left(z_{1},z_{2},z_{3}\right)}_{l},{\left(w_{1},w_{2},w_{3}\right)}_{l}\right\}}_{l=1}^{50}$ corresponding to these paths. Following Example 1, we estimate the covariance functions ${\hat{\Sigma }}_{X}$ and ${\hat{\Sigma }}_{Y}$ using the respective coefficients and plug this covariance matrix into the Shannon entropy expression to obtain the estimated entropies ${\hat{H}}_{1}\left(X\right)=1.402$ and ${\hat{H}}_{2}\left(Y\right)=99.552$, similar to the true entropies $H_{1}\left(X\right)=1.428$ and $H_{2}\left(Y\right)=91.420$, respectively. We formally propose the estimation procedure in Algorithm 1.

 **Algorithm 1:** Estimation of $H_{\alpha }\left(X,K\right)$ from a sample of random paths. 
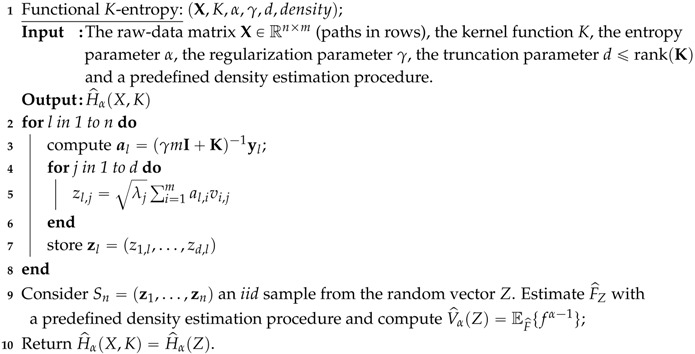


The choice of kernel parameters in Algorithm 1 is made by cross-validation. This ensures that the curve fitting method is asymptotically optimal. Nonetheless, although the selection of the kernel parameters affects the scale of the estimated entropy, the center-outward ordering induced by $H_{\alpha }\left(X,K\right)$, as formally proposed in the next section, is unaffected. In the Supplementary Material, we present relevant experimental results to illustrate this property, which make the method robust in terms of the selection of the kernel and regularization parameters.

## 3. Minimum Entropy for Anomaly Detection

Anomaly detection is a common task in almost all data analysis context. The unsupervised approach considers a sample $X_{1},\ldots ,X_{n}$ of random elements where most instances follow a well-defined pattern and a small proportion, here denoted as ν∈[0,1], present an abnormal pattern. In recent works (see for instance [10,11,12,13]), the authors propose depth measures and related methods, to deal with functional outliers. In this section, we propose a novel criterion to tackle the problem of anomaly detection with functional data using the ideas and concepts developed in Section 2. For a real-valued *d*-dimensional random vector *Z* that admits a continuous density function $f_{Z}$, define $H_{\alpha }\left(A_{Z}\right)=\frac{1}{1-\alpha }\log\left(\int_{A}f_{Z}^{\alpha }\left(z\right)dz\right)$ to be the entropy of the Borel-set *A* with respect to the measure $F_{Z}$. Then, the ν-Minimal-Entropy Set (MES) is formally defined as:

$${\mathrm{MES}}_{\nu }\left(Z\right):=\left\{{arg min}_{A\subset R^{d}}H_{\alpha }\left(A_{Z}\right)\;s.t.\;P\left(A\right)\geq 1-\nu \right\}.$$

The ${\mathrm{MES}}_{\nu }$ is equivalent [14,15] to a ν-High Density Set (HDS) [16] formally defined as ${\mathrm{HDS}}_{\nu }\left(Z\right)=\left\{z\in R^{d}|\;f_{Z}\left(z\right)>c_{\nu }\right\}$, where $c_{\nu }$ is the largest constant such that $P({\mathrm{HDS}}_{\nu }\left(Z\right))\geq 1-\nu$, for 0<ν<1. Therefore, the complement of MES is a suitable set to define outlier data in the sample, considering $\tilde{x}\left(t\right)\notin {\mathrm{MES}}_{\nu }$ as an atypical realization of *X*. Next, we give two approaches to estimate MES.

### 3.1. Parametric Approach

Given a random sample of *n* discrete random paths $\left\{x_{1}\left(t_{i}\right),\ldots ,x_{n}\left(t_{i}\right)\right\}$ for i=1,…,m, we transform this sample into *d*-dimensional vectors $S_{n}=\left(z_{1},\ldots ,z_{n}\right)$ using the representation and truncation method proposed in this work, numerically implemented in Lines 2–8 in Algorithm 1. Assume further that $f_{Z}\left(z,\theta \right)$ is a suitable probability model for the random sample $z_{1},\ldots ,z_{n}$, then we estimate by Robust Maximum Likelihood (RML) the parameters θ. For instance, in this paper, we consider $f_{Z}\left(z,\theta \right)$ to be the normal density, and then, RML estimated parameters are $\hat{\theta }=\left(\hat{\mu },\hat{\Sigma }\right)$, the robust mean vector and covariance matrix, respectively. For details on robust estimation, we refer to [17]. After the estimation of the distribution parameters, the computation of $H_{\alpha }$ follows by plugging the estimated density $f_{Z}\left(z,\hat{\theta }\right)$ into Equation (1). Moreover, for the normal model, the estimated set ${\mathrm{MES}}_{\nu }$ is defined trough the following expression:

$${\mathrm{MES}}_{\nu }\left(S_{n}\right)=\left\{z\in R^{d}|{\left(z-\hat{\mu }\right)}^{T}{\hat{\Sigma }}^{-1}\left(z-\hat{\mu }\right)\leq \chi_{d}^{2}\left(\nu \right)\right\},$$

where $\chi_{d}^{2}\left(\nu \right)$ is the 1−ν quantile of a Chi-square distribution with *d*-degrees of freedom. Then, if the coefficient $z_{i}$, representing ${\tilde{x}}_{i}\left(t\right)$, lies outside this ellipsoid, we say that the functional datum is atypical. When the proportion of outlier ν in the sample is known a priori, the $\chi_{d}^{2}\left(\nu \right)$-quantile can be replaced by the corresponding sample 1−ν Mahalanobis distance quantile, as is the case in Section 4.1.

### 3.2. Non-Parametric Approach

The following are definitions to introduce further non-parametric estimation methods. For the random vector $Z\in R^{d}$ distributed according to $F_{Z}$, let $B_{Z}\left(z,r_{\delta }\right)\subset R^{d}$ be the z-centered ball with radius $r_{\delta }$ that fulfills the condition $\delta =\int_{B_{Z}\left(z,r_{\delta }\right)}f_{Z}\left(z\right)dz$, then the δ-neighbors of the point z comprise the open set $\Delta_{z}=R^{d}⋂B\left(z,r_{\delta }\right)$.

**Definition** **3** (*δ*-local *α*-entropy)**.** *Let $z\in R^{d}$, for α>0 and α≠1; the δ-local α-entropy of the r.v. Z is:*

$$h_{\alpha }\left(\Delta_{z}\right)=\frac{1}{1-\alpha }\log\left(\int_{\Delta_{z}}f_{Z}^{\alpha }\left(z\right)dz\right)\;for\;all\;z\in R^{d}.$$

Under mild regularity conditions on $f_{Z}$, the local entropy measure is a suitable metric to characterize the degree of abnormality of every point z in the support of $F_{Z}$. Several natural estimators of local entropy measures can be considered, for instance the (average) distance from the point z to its *k*-th-nearest neighbor. We estimate MES combining the estimated δ-Local α-entropy. As in the parametric case, let $\left\{x_{1}\left(t_{i}\right),\ldots ,x_{n}\left(t_{i}\right)\right\}$ for i=1,…,m be a random sample of *n* discrete random paths; we transform this sample into *d*-dimensional vectors $S_{n}=\left(z_{1},\ldots ,z_{n}\right)$ following Lines 2–8 in Algorithm 1. Next, we estimate the local entropy for these data using the estimator ${\hat{h}}_{\alpha }\left(\Delta_{z_{i}}\right)=\exp\left({\bar{d}}_{k}\left(z_{i},S_{n}\right)\right)$, where ${\bar{d}}_{k}\left(z_{i},S_{n}\right)$ is the average distance from $z_{i}$ to its *k*-th-nearest neighbor [18], and then estimate ${\mathrm{MES}}_{\nu }$ solving the following optimization problem:

(8) $$\max_{\rho ,\epsilon_{1},\ldots ,\epsilon_{n}}\left(1-\nu \right)\rho -\frac{1}{n}\sum_{i=1}^{n}\epsilon_{i}\;s.t.\;{\hat{h}}_{\alpha }\left(\Delta_{z_{i}}\right)\geq \rho -\epsilon_{i},\;\epsilon_{i}\geq 0\;\mathrm{for}\;i=1,\ldots ,n.$$

The solution to this problem, $\rho^{*}$, leads to the following decision function:

$$D\left(z\right)=\mathrm{sign}(\rho^{*}-{\hat{h}}_{\alpha }\left(\Delta_{z}\right)),$$

where D(z)=+1 if z corresponds to the (1−ν) proportion of curves projected near the origin, that is the set of curves that belongs to a low entropy (high density) set. The following theorem shows that as the number of available curves increases, the estimation method asymptotically detects the proportion 1−ν of curves belonging to the ${\mathrm{MES}}_{\nu }$.

**Theorem** **1.** *At the solution of the optimization problem stated in Equation 8, the following equality holds:*

$$\lim_{n\to \infty }\frac{1}{n}\sum_{i=1}^{n}I\left(z_{i}\right)=1-\nu ,$$

where I(z)=1 if ${\hat{h}}_{\alpha }\left(\Delta_{z}\right)\leq \rho^{*}$ and I(z)=0 otherwise.

## 4. Experimental Section

The aim of this section is to illustrate the performance of the proposed methodology to detect abnormal observations in a sample of functional data. In what follows, for the representation of functional data, we consider the Gaussian kernel function $K\left(t_{l},t_{k}\right)=e^{-\sigma ∥t_{l}-t_{k}{∥}^{2}}$. The kernel parameter σ and the regularization coefficient γ in Algorithm 1 were defined through cross-validation.

### 4.1. Simulation Analysis

In a Monte Carlo study, we investigate the performance of the proposed method over three data configurations (Scenarios A, B and C). Specifically, we consider the following generating processes: a fraction 1−ν of n=400 curves are realizations of the following stochastic model:

$$X_{l}\left(t\right)=\sum_{j=1}^{4}\xi_{j}\sin\left(j\pi t\right)+\varepsilon_{l}\left(t\right),\;\mathrm{for}\;l=1,\ldots ,\left(1-\nu \right)n,\;\mathrm{and}\;t\in \left[0,1\right],$$

where $\xi =(\xi_{1},\ldots ,\xi_{4})$ is a normally-distributed multivariate random variable with mean $\mu_{\xi }=\left(4,2,4,1\right)$ and diagonal co-variance matrix $\Sigma_{\xi }=\mathrm{diag}\left(5,2,2,1\right)$, and $\varepsilon_{l}\left(t\right)$ are independent autocorrelated random error functions.

The remaining proportion of data nν with ν∈{1%,5%,10%} comprises outliers that contaminate the sample according to the following typical scenarios (see [19]):

(A) Magnitude outliers: $Y_{l}\left(t\right)=\sum_{j=1}^{4}\zeta_{j}\sin\left(j\pi t\right)+\varepsilon_{l}\left(t\right),\;\mathrm{for}\;l=1,\ldots ,\nu n,\;\mathrm{and}\;t\in \left[0,1\right],$ where ζ is a normally-distributed multivariate r.v. with parameters $\mu_{\zeta }=2.5\mu_{\xi }$ and $\Sigma_{\zeta }={\left(2.5\right)}^{2}\Sigma_{\xi }$.
(B) Shape outliers: $Y_{l}\left(t\right)=\sum_{j=1}^{4}\zeta_{j}\sin\left(j\pi t\right)+\varepsilon_{l}\left(t\right),\;\mathrm{for}\;l=1,\ldots ,\nu n,\;\mathrm{and}\;t\in \left[0,1\right],$ where ζ is a normally-distributed multivariate r.v. with parameters $\mu_{\zeta }=\left(4,-2,1,3\right)$ and $\Sigma_{\zeta }=\Sigma_{\xi }$.
(C) A combination considering νn/2 outliers from Scenario A and νn/2 outliers from Scenario B.

**Figure 2:**
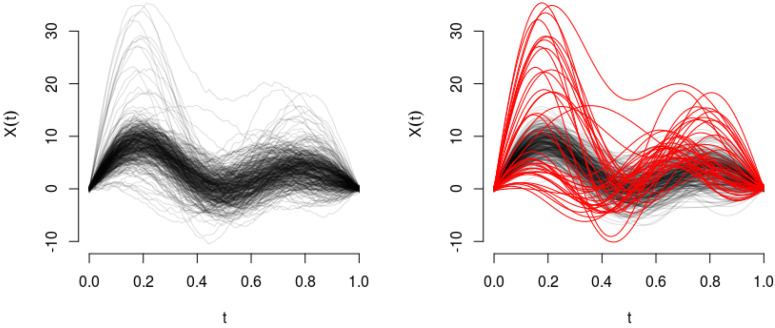
(Left) Raw data, 400 curves corresponding to Scenario C with ν=10%. (Right) Functional data, in black (“—”), the sample of regular paths X(t), and abnormal curves Y(t) in red (“—”).

To illustrate the generating process, in Figure 2, we show one instance of the simulated paths in Scenario C with ν=10%. We test our Parametric entropy (PA) and Non-Parametric entropy (NPA) method against several well-known depth measures for functional anomaly detection, namely: the Modified Band Depth (MBD), the H-Mode Depth (HMD), the Random Tukey Depth (RTD) and the Functional Spatial Depth (FSD) (see [10,11,12,13]), respectively, already implemented in the R-package fda-usc [20]. For this experiment, the values of the parameter ν are assumed known in each scenario. With respect to parameters σ and γ in Algorithm 1, in this simulation exercise, we chose them with a 10-fold cross-validation procedure using a single set of data, which correspond to the first instance of the simulations. The reference values (which remain fixed throughout the simulation exercise) are σ=10 and $\gamma =0.1^{5}$.

Let P and N be the amount of outlier and normal data in the sample, respectively, and let TP = True Positive and TN = True Negative be the respective quantities detected by different methods; in Table 1, we report the following average metrics TPR = TP/P (True Positive Rate or sensitivity), TNR = TN/N (True Negative Rate or specificity) and the area under the ROC curve (aROC) of each method obtained through the M=1000 replications in the Monte Carlo study.

As can be seen, the PA and NPA entropy methods proposed in this article outperform other recently-proposed depth measures in the three scenarios considered in the experiments when ν={0.10,0.05}. In the remaining case (when ν=0.01), PA and NPA outperform the other methods; however, the standard errors are slightly high to confirm a significant difference between the methods.

When we compare among the proposed methods, the parametric approach seems to be slightly (but consistently) more effective than the non-parametric approach in Scenario A. For Scenarios B and C, both methods provide similar results. It is important to remark that the PA method is especially adequate for Gaussian data, while the NPA method does not assume any distributional hypothesis on the data. In this sense, the simulation results show the robustness of the non-parametric approach even when competing with parametric methods designed for specific distributions.

### 4.2. Outliers in the Context of Mortality-Rate Curve Analysis

**Figure 3:**
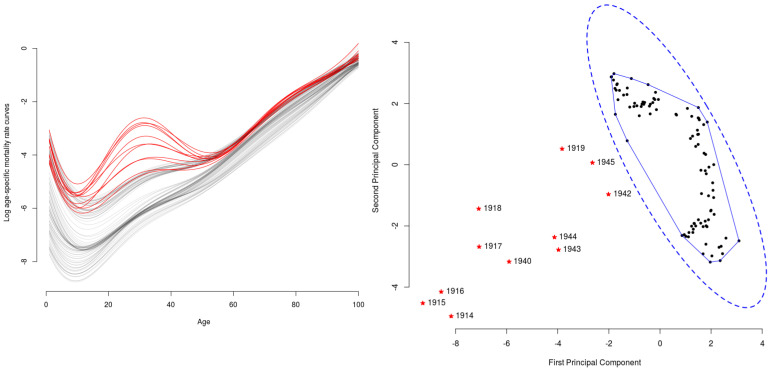
French mortality data: On the left, the regular curves in black (“—”) and outliers detected in red (“—”) for ν=10%. On the right, the first two principal components of the kernel eigenfunctions; the area inside the doted blue ellipsoid (- -) corresponds PA estimation of ${\mathrm{MES}}_{\nu =90\%}$ and the region inside the convex hull in blue (—) to the NPA estimation. The regular curves, represented with black dots (•), lie inside the ${\mathrm{MES}}_{\nu =90\%}$ and detected outliers with a red asterisk (∗) outside of ${\mathrm{MES}}_{\nu =90\%}$.

We consider the French mortality rates database, available in the R-package Demography [21], to study age-specific male death rates in a logarithmic scale. In Figure 3 (left), each curve corresponds to one year from 1901–2006 (106 paths in total) and accounts for the number of deaths per 1000 of the mean population in the age group (from 0–101 years) in question. As expected, for low-age cohorts (until 12 years, approximately), the mortality rates present a decreasing trend and then start to grow until late ages, where all cohorts achieve a 100% mortality rate.

For some years, the evolution pattern of mortality presents an atypical behavior, mostly coinciding with the first and second World Wars, jointly with the influenza pandemic episode that took place in 1919.

In this experiment, we do not know a priori the proportion of atypical curves. Therefore, after having conducted inference over a wide range of values for ν, as a way to assess the sensitivity and reliability of the inference when determining the number of abnormal curves, we decided to fix ν=10%. For further details on the way to choose the parameter ν (and an extended sensitivity analysis on the values of ν), please refer to § 3.2 in the Supplementary Material. In Figure 3 (left), we highlight in red the anomalous detected curves with both the entropy-PA and NPA methods corresponding to the years 1914–1919 and 1940, 1942–1945, which match with men (between 20 and 40 years old) participating in World War I and II. In Figure 3 (right), we use the first two principal components of the kernel eigenfunctions to project the representation coefficients (in this experiment, in $R^{14}$) in two dimensions. As can be seen, the points laying outside the ${\mathrm{MES}}_{\nu =90\%}$, represented with doted-blue ellipses when estimating it with PA (- -) and the convex hull with a continuous blue line (—) when estimating it with NPA, correspond to the the atypical curves in the sample.

## 5. Discussion

In this article, we propose a definition of entropy for stochastic processes. We provide a reproducing kernel Hilbert space model to estimate entropy from a random sample of realizations of a stochastic process, namely functional data, and introduce two approaches to estimate minimum entropy sets for functional anomaly detection.

In the experimental section, the Monte Carlo simulation illustrates the adequacy of the proposed method in the context of magnitude and shape outliers, outperforming other state of the art methods for functional anomaly detection. In the study of French mortality rates, the parametric and non-parametric approaches for minimum entropy sets estimation show their adequacy to capture anomalous curves, principally associated with the First and Second World Wars and the Influenza episode in 1919.

Regardless of the results presented in the paper, how widely the method can be used in practice, especially with noisier data, is an open question. In this sense, as future work, we will consider testing the performance of the proposed method in other scenarios with different noise assumptions in the observations. Another natural extension for future work entails the study of the asymptotic properties of the ${\mathrm{MES}}_{\nu }$ estimators. The extension of the proposed method from the stochastic process to random fields, useful for several statistical and information science areas, seems straightforward, but a wide range of simulations and numerical experiments must be done in order to stress the performance of entropy methods in comparison to other techniques when dealing with abnormal fields. Another natural avenue for future work entails the study of the connections between entropy for stochastic process, as formally defined here, and the maximum entropy principle when estimating the governing parameters of Gaussian processes.

## Figures and Tables

**Table 1:** Simulation analysis: Scenarios and contamination percentages ν in columns. In rows, different methods and average sensitivities, specificities and the areas under the ROC curves (aROC) (this last on a scale of 10^2^). The corresponding standard-error is reported in parenthesis.

Method	Metric	Scenario A			Scenario B			Scenario C		
		10%	5%	1%	10%	5%	1%	10%	5%	1%
MBD	TPR	74.867	71.010	55.300	48.275	39.395	13.475	67.787	58.365	36.300
		(4.699)	(7.712)	(20.852)	(5.914)	(9.013)	(16.180)	(5.351)	(7.772)	(18.341)
	TNR	97.207	98.474	99.548	94.252	96.810	99.126	96.420	97.808	99.356
		(0.522)	(0.406)	(0.210)	(0.657)	(0.474)	(0.163)	(0.594)	(0.409)	(0.185)
	aROC	96.662	97.375	97.735	89.393	91.693	93.244	95.272	95.444	95.354
		(1.245)	(1.517)	(3.059)	(2.033)	(2.388)	(4.425)	(1.399)	(1.831)	(4.370)
HMD	TPR	92.665	91.545	88.675	66.532	62.780	47.475	79.992	76.765	66.025
		(3.295)	(5.173)	(14.793)	(6.084)	(8.809)	(21.206)	(4.562)	(7.039)	(18.004)
	TNR	99.185	99.555	99.885	96.281	98.041	99.469	97.776	98.777	99.656
		(0.366)	(0.272)	(0.149)	(0.676)	(0.463)	(0.214)	(0.506)	(0.370)	(0.181)
	aROC	99.200	99.256	99.346	94.980	96.153	96.969	97.676	97.924	97.842
		(0.851)	(1.105)	(2.391)	(1.583)	(1.812)	(3.473)	(1.089)	(1.401)	(3.542)
RTD	TPR	83.555	83.045	76.400	50.972	43.940	22.700	71.975	65.225	49.700
		(4.743)	(0.694)	(18.931)	(9.409)	(1.279)	(2.1334)	(7.178)	(9.716)	(1.834)
	TNR	98.174	99.104	99.762	94.544	97.049	99.218	96.889	98.165	99.491
		(0.526)	(0.365)	(0.191)	(1.045)	(0.674)	(0.215)	(0.798)	(0.511)	(0.184)
	aROC	98.187	98.605	98.962	90.426	92.510	94.154	96.156	96.345	96.242
		(1.094)	(1.347)	(2.538)	(2.817)	(2.967)	(4.574)	(1.580)	(1.977)	(4.085)
FSD	TPR	81.472	83.215	81.925	50.275	46.550	27.400	74.775	69.485	53.775
		(3.978)	(5.947)	(16.671)	(5.238)	(8.018)	(19.547)	(4.601)	(6.859)	(16.707)
	TNR	97.941	99.116	99.817	94.475	97.186	99.267	97.197	98.396	99.533
		(0.442)	(0.313)	(0.168)	(0.582)	(0.421)	(0.197)	(0.511)	(0.361)	(0.168)
	aROC	97.934	98.738	99.163	90.059	93.279	95.485	96.777	97.148	97.125
		(1.030)	(1.232)	(2.490)	(1.794)	(2.061)	(3.723)	(1.158)	(1.477)	(3.682)
Entropy-PA	TPR	**94.150**	**93.215**	**91.725**	**80.740**	**77.390**	66.925	**87.550**	84.935	77.650
		(3.078)	(4.817)	(12.591)	(6.250)	(8.550)	(20.330)	(4.632)	(6.604)	(17.015)
	TNR	**99.350**	**99.649**	**99.916**	**97.860**	**98.810**	99.664	**98.616**	99.207	99.774
		(0.342)	(0.253)	(0.127)	(0.694)	(0.450)	(0.205)	(0.514)	(0.347)	(0.171)
	aROC	**99.351**	**99.353**	**99.374**	**97.549**	97.987	98.301	98.677	98.752	98.641
		(0.788)	(1.078)	(2.474)	(1.364)	(1.495)	(2.785)	(0.944)	(1.208)	(3.081)
Entropy-NPA	TPR	92.725	91.505	89.050	74.215	77.145	**71.250**	87.225	**85.805**	**79.775**
		(3.325)	(5.228)	(14.630)	(6.237)	(7.904)	(19.970)	(4.217)	(6.198)	(16.788)
	TNR	99.191	99.552	99.889	97.135	98.792	**99.709**	98.586	**99.252**	**99.795**
		(0.369)	(0.275)	(0.147)	(0.693)	(0.416)	(0.201)	(0.468)	(0.326)	(0.169)
	aROC	99.243	99.266	99.293	97.240	**98.253**	**98.685**	**98.782**	**98.880**	**98.861**
		(0.815)	(1.097)	(2.528)	(1.130)	(1.250)	(2.550)	(0.856)	(1.145)	(2.880)

## Supplementary Materials

The following are available online at www.mdpi.com/1099-4300/20/1/33/s1.

## Abbreviations

The following abbreviations are used in this manuscript:

RML	Robust Maximum Likelihood.
MES and HDS	Minimum Entropy and High Density Sets, respectively.
PA and NPA	Parametric and Non-Parametric approaches.
MBD, HMD, RTD, FSD	Modified Band, H-Mode, Random Tukey and Functional Spatial Depths.

## Appendix A

**Proof** **Theorem** **1.** Consider the following optimization problem:

(A1) $$\min_{\beta_{1},\ldots ,\beta_{n}}\sum_{i=1}^{n}\beta_{i}{\hat{h}}_{\alpha }\left(\Delta_{z_{i}}\right)\;s.t.\;\sum_{i=1}^{n}\beta_{i}=n\left(1-\nu \right)\;\;\mathrm{and}\;\;0\leq \beta_{i}\leq 1\;\mathrm{for}\;i=1,\ldots ,n.$$

For the sake of simplicity, consider first the case where n(1−ν)∈N. Let $q^{*}$ be the 1−ν quantile of the $S_{n}$ sample. Then, it can be shown that $\beta_{i}^{*}=1$ if ${\hat{h}}_{\alpha }\left(\Delta_{z_{i}}\right)\leq q^{*}$ and $\beta_{i}^{*}=0$ if ${\hat{h}}_{\alpha }\left(\Delta_{z_{i}}\right)>q^{*}$ is a solution for the problem stated in Equation (A1). As a consequence:

$$\frac{1}{n}\sum_{i=1}^{n}I\left(z_{i}\right)=\frac{1}{n}\sum_{i=1}^{n}\beta_{i}^{*}.$$

From the constraint in Equation (A1), it holds that $\sum_{i=1}^{n}\beta_{i}^{*}=n\left(1-\nu \right)$, and then:

$$\lim_{n\to \infty }\frac{1}{n}\sum_{i=1}^{n}\beta_{i}^{*}=\lim_{n\to \infty }\frac{1}{n}n\left(1-\nu \right)=1-\nu$$

For the case n(1−ν)∉N, it holds that $\left\{\begin{array}{cc} \beta_{i}=1, & \mathrm{if}\;\;\;{\hat{h}}_{\alpha }\left(\Delta_{z_{i}}\right)<q^{*}\\ \beta_{i}=n\left(1-\nu \right)-\left[n\left(1-\nu \right)\right], & \mathrm{if}\;\;\;{\hat{h}}_{\alpha }\left(\Delta_{z}\right)=q^{*}\\ \beta_{i}=0, & \mathrm{if}\;\;\;{\hat{h}}_{\alpha }\left(\Delta_{z_{i}}\right)>q^{*} \end{array}\right.$ where [z] stands for the largest integer no greater than *x*. Therefore, the number of $\beta_{i}^{*}$’s equating to one is [n(1−ν)] and:

$$\lim_{n\to \infty }\frac{1}{n}\sum_{i=1}^{n}I\left(z_{i}\right)=\lim_{n\to \infty }\frac{1}{n}\left(\left[n\left(1-\nu \right)\right]\times 1+1\right)=\lim_{n\to \infty }\frac{\left[n(1-\nu )\right]}{n}=1-\nu .$$

Finally, we show that $\rho^{*}=q^{*}$. The dual problem of (A1) is:

(A2) $$\max_{b,\epsilon_{1},\ldots ,\epsilon_{n}}n\left(1-\nu \right)b-\sum_{i=1}^{n}\epsilon_{i}\;s.t.\;{\hat{h}}_{\alpha }\left(\Delta_{z_{i}}\right)\geq b-\epsilon_{i},\;\epsilon_{i}\geq 0\;\mathrm{for}\;i=1,\ldots ,n.$$

By the fundamental theorem of duality, the objective functions of the problems stated in Equations (A1) and (A2) take the same value at their solutions, and as a consequence, $b^{*}=q^{*}$ (see [22]). Since Problem (A2) differs from Problem (8) just in the scaling of the objective function, it holds that $\rho^{*}=b^{*}$, which concludes the proof. ☐
