# Supplementary material for: Entropy Measures for Stochastic Processes with Applications in Functional Anomaly Detection
Source: Entropy (Basel). 2018 Jan 11;20(1):33. doi: 10.3390/e20010033 (PMC7512230; doi:10.3390/e20010033)
Supplement: Supplementary file 1 [file entropy-20-00033-s001.pdf]

Article

Supplementary Material: Entropy Measures for Stochastic Processes with Applications in Functional Anomaly Detection

Gabriel Martos [1,*], Nicolás Hernández [2], Alberto Muñoz [2] and Javier M. Moguerza [3]

[1] Universidad de Buenos Aires and CONICET; gabriel.martos@ic.fcen.uba.ar
[2] Universidad Carlos III de Madrid; {nihernan,albmun}@est-econ.uc3m.es
[3] Universidad Rey Juan Carlos; javier.moguerza@urjc.es

Academic Editor: name
Version January 4, 2018 submitted to Entropy

1. A Numerical Study of the Convergence of the Estimated Entropy

The aim of this section is to show the convergence of the entropy estimation under the parametric approach through a Monte Carlo experiment. To this end, we consider the Gaussian processes of Example 1: $X(t) = \sum_{i=1}^{3} \xi_i e_i(t)$ and $Y(t) = \sum_{i=1}^{3} \zeta_i e_i(t)$; where $e_i(t)$ is a Fourier basis in $T = [0,1]$, $\xi_i \sim N(\mu = 0, \sigma^2 = 0.5)$, and $\zeta_i \sim N(\mu = 0, \sigma^2 = 2)$ are independent normally-distributed r.v. for $i = 1,2,3$.

The Shannon-entropies ($\alpha = 1$) of these two stochastic processes are as follows:

$$H_1(X, d = 3) = \frac{1}{2}\log(2\pi e)^3 \det(\mathbf{\Sigma}_X) \text{ and } H_1(Y, d = 3) = \frac{1}{2}\log(2\pi e)^3 \det(\mathbf{\Sigma}_Y),$$

where $\mathbf{\Sigma}_X$ and $\mathbf{\Sigma}_Y$ are the respective covariance matrices. The Monte Carlo experiment was carried out with $M = 10{,}000$ samples from the distribution of ξ_i and ζ_i, for different sample sizes $N = \{5, 10, 50, 100, 200, 500, 750, 1000, 1500, 2000, 3000\}$. The results, illustrated in Figure 1, show that in both cases, the estimated Shannon-entropy converges relatively fast to the true values, $H_1(X) = 1.428$ and $H_2(Y) = 91.420$.

2. Order Invariance Property and Robustness

The entropy measure of a stochastic process is a 'K-entropy', which means that the estimated entropy depends on the choice of a particular kernel. In this sense, is the order in the sampled curves (from most to least depth curves) induced by the entropy measure invariant to changes in the kernel function? What we numerically show next is that the order induced by the entropy does not depend on the the kernel function (or its parameters) when representing the functional data at hand. To illustrate this, we constructed an experiment considering Scenario A in Section 4.1 of the paper when $n = 1000$ and $\nu = 0.05$. As the aim of this section is to show the order invariance property, we consider two different kernel function and different parameters, namely:

i) The Gaussian kernel function:

$$K_G(t_l, t_k) = e^{-\sigma\|t_l - t_k\|^2}, \text{with } \sigma = 5, 10, 15.$$

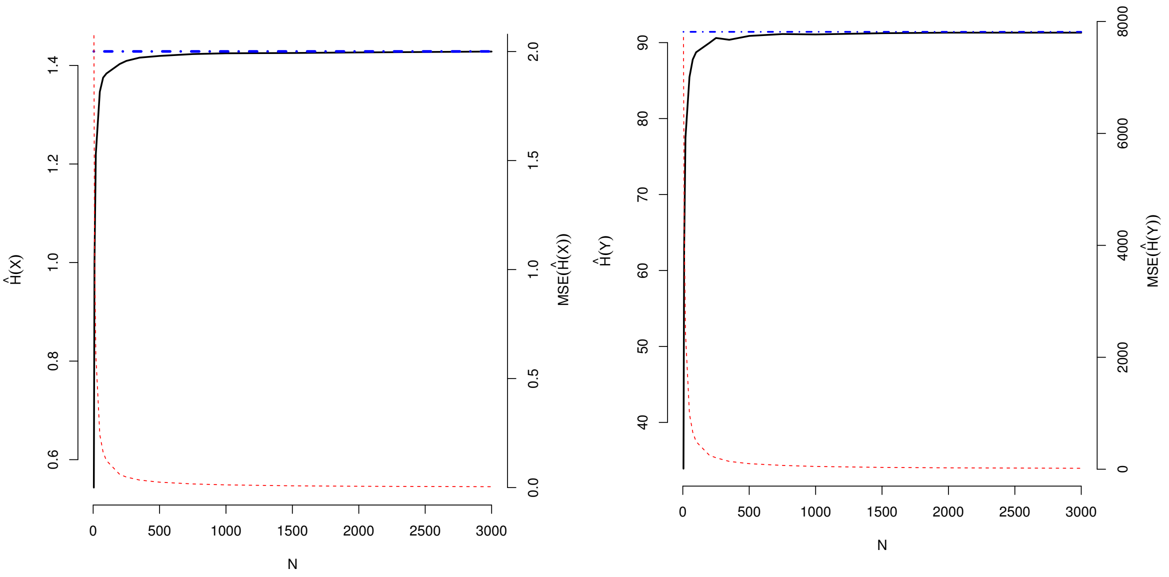

Figure 1. Entropy estimation in black ("—"), entropy true value in blue ("- · -") and mean squared error in red ("- -") for the two Gaussian processes $X(t)$ (left) and $Y(t)$ (right).

ii) The spline kernel function:

$$K_S(t_l, t_k) = \prod_{d=1}^{D} 1 + t_l t_k + t_l t_k \min(t_l, t_k) - \frac{t_l + t_k}{2} \min(t_l, t_k)^2 + \frac{t_l + t_k}{3} \min(t_l, t_k)^3.$$

The results, displayed in Figure 2 in the case of the parametric approach and Figure 3 for the non-parametric approach, show that the order induced in the sample curves by the entropy measure is invariant to changes in the kernel function considered. This property makes the method robust in terms of the selection of the kernel and regularization parameters. This exercise was also carried out for different sample sizes, $N = \{2000, 3000, 5000\}$ and different values of parameter ν, with similar results (the R code to replicate the experiment is available upon request).

3. Supplementary Empirical Report

3.1. An Additional Single Run Simulation Study

The aim of this experiment is to illustrate the performance of the proposed methodology when the atypical data cannot be inferred considering particular extreme points in the curves and under different assumptions about the noise in the observed data. To this aim, a fraction $1 - \nu = 90\%$ of $n = 400$ curves comprises the realizations of the following stochastic model:

$$X_l(t) = \sin(t) + \cos(t + \varepsilon_l) + a_l + b_l t^2, \text{ for } l = 1, \ldots, (1-\nu)n, \text{ and } t \in [0, 2\pi],$$

where the random coefficients $(\varepsilon_l, a_l, b_l)$ are independently and normally distributed with means: $\mu_\varepsilon = 0$, $\mu_a = 5$ and $\mu_b = 1$ and variances $\sigma_\varepsilon^2 = \sigma_b^2 = 0.25$ and $\sigma_a^2 = 0.2$. The remaining proportion of the data comprises outliers that contaminate the sample according to the following stochastic model:

$$Y_l(t) = \sin(t) + \cos(t + \varepsilon_l) + \frac{1}{2}(\sin(2\pi t) + \cos(\pi t + \varepsilon_l)) + a_l + b_l t^2, \text{ for } l = 1, \ldots, n\nu, \text{ and } t \in [0, 2\pi],$$

where the random coefficients $(\varepsilon_l, a_l, b_l)$ are independently and normally distributed with the same means and variances as in the case of $X(t)$. In Figure 4, we show simulated raw data on the left and

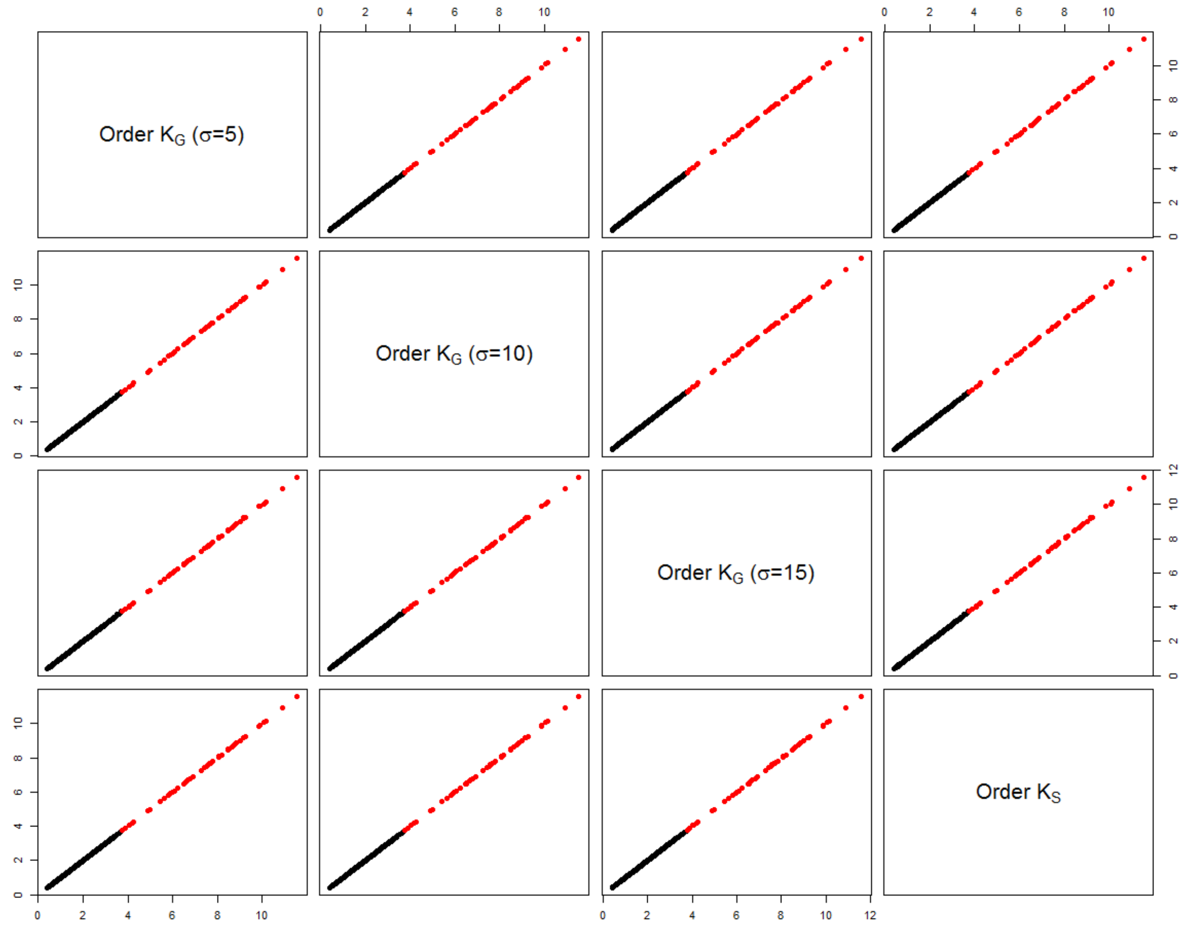

Figure 2. Order induced by the entropy estimation (parametric approach) for different kernel functions, with $\nu = 5\%$. The regular curves, corresponding to $X(t)$, in (•) and the detected outliers, corresponding to $Y(t)$, in (•).

the corresponding functional data on the right, as in the paper, we use a Gaussian kernel and choose the parameters by cross-validation.

In Figure 5, we illustrate the outliers captured with the proposed method in red (—), false positives in blue (—) and false negatives in green (—). The parametric approach (Figure 5 (left)) captures all the atypical curves in the sample without any false positive, nor false negative finding. The non-parametric approach (Figure 5 (right)) shows slightly worse performance incurring four false positive detections and four false negative occurrences. In Table1, we report the TPR, the TNR and the aROC; as can be seen, the proposed methods clearly outperform the other methods in the literature.

Table 1. Different methods sensitivities, specificities and precisions.

Method	TPR	TNR	aROC
MBD	5.0	89.4	0.452
HMD	12.5	90.3	0.701
RTD	10.0	90.0	0.591
FSD	7.5	89.7	0.645
Entropy-PA	**100.0**	**100.0**	**1.000**
Entropy-NPA	90.0	98.9	0.992

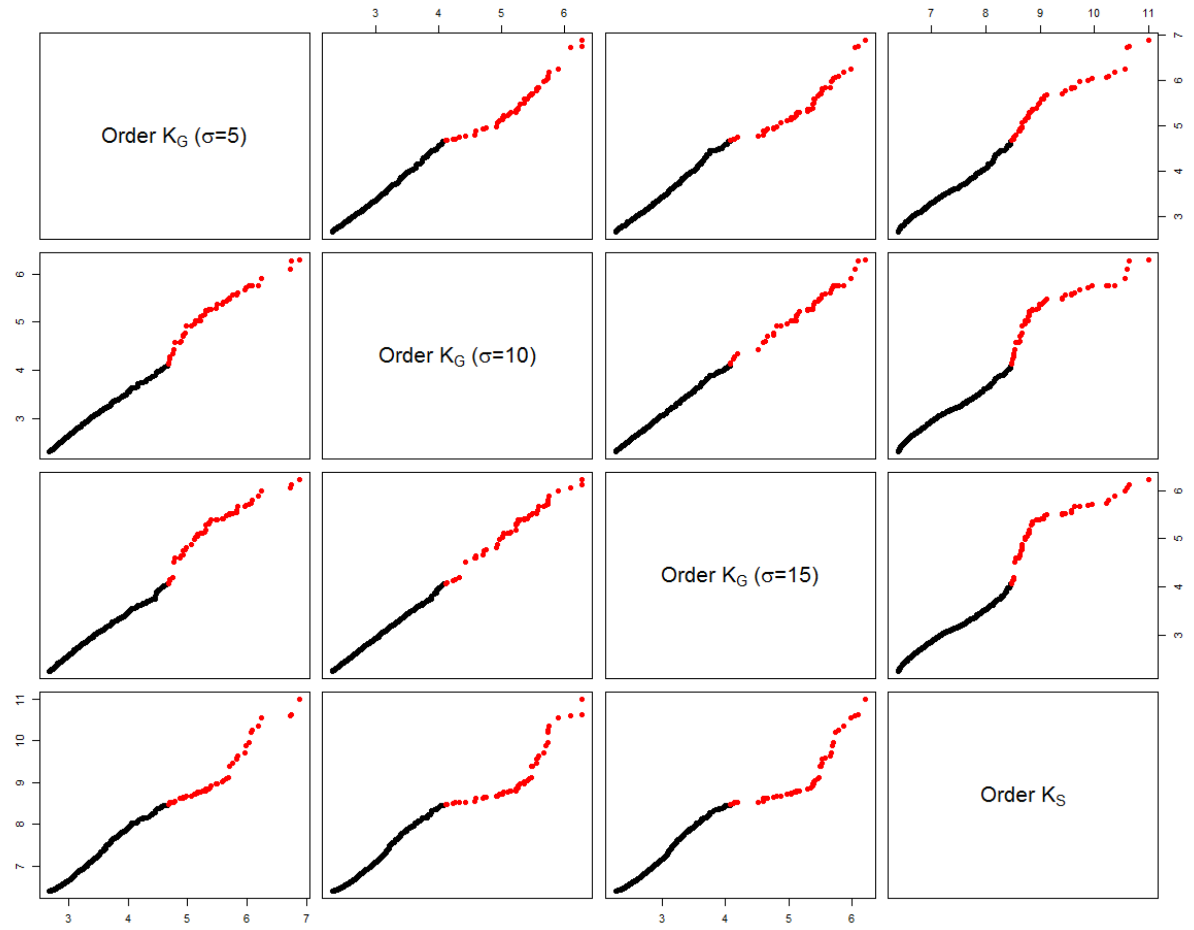

Figure 3. Order induced by the entropy estimation (non-parametric approach) for different kernel functions, with $\nu = 5\%$. The regular curves, corresponding to $X(t)$, in (•) and the detected outliers, corresponding to $Y(t)$, in (•).

3.2. Outliers in the Context of Mortality Rate Curve Extended Analysis

In this section, we present an extended analysis of the empirical exercise of outlier detection in the context of mortality rate curves. In Table 2, we present the full results of the anomaly detection exercise considering entropy-PA and entropy-NPA and the results obtained with other measures described in Section 4 for $\nu = \{0.50, 0.25, 0.15, 0.10, 0.05, 0.01\}$. In the first three scenarios, that is when $\nu = \{0.50, 0.25, 0.15\}$, the results for the competitor measures show that only the HMD is able to capture almost all curves corresponding to the First and Second World War (except year 1941) and the influenza pandemic for a value of $\nu = 0.25$. As is expected, the use of an inappropriate value for ν increases the number of false positives in the analysis. A convenient criterion for choosing the value of ν is to consider the ratio:

$$D_M(\mathbf{z}_{[i]}, \widehat{\boldsymbol{\mu}}_z) / \sum_{i=1}^{n} D_M(\mathbf{z}_{[i]}, \widehat{\boldsymbol{\mu}}_z),$$

where $D_M(\mathbf{z}_{[i]}, \widehat{\boldsymbol{\mu}}_z)$ represents the Mahalanobis distance sorted in deceasing order of the vector $\mathbf{z}_{[i]}$ representing a curve in the sample (in the case of non-parametric approach, we consider the sorted sequence of estimated local entropies). Using this criterion, in Section 4.2, we have decided to fix $\nu = 10\%$, since, as can be seen in Figure 6, the distributions of the estimated robust Mahalanobis distances (left) and the local entropies (right) show an elbow at Points 10 and 4 respectively, and this

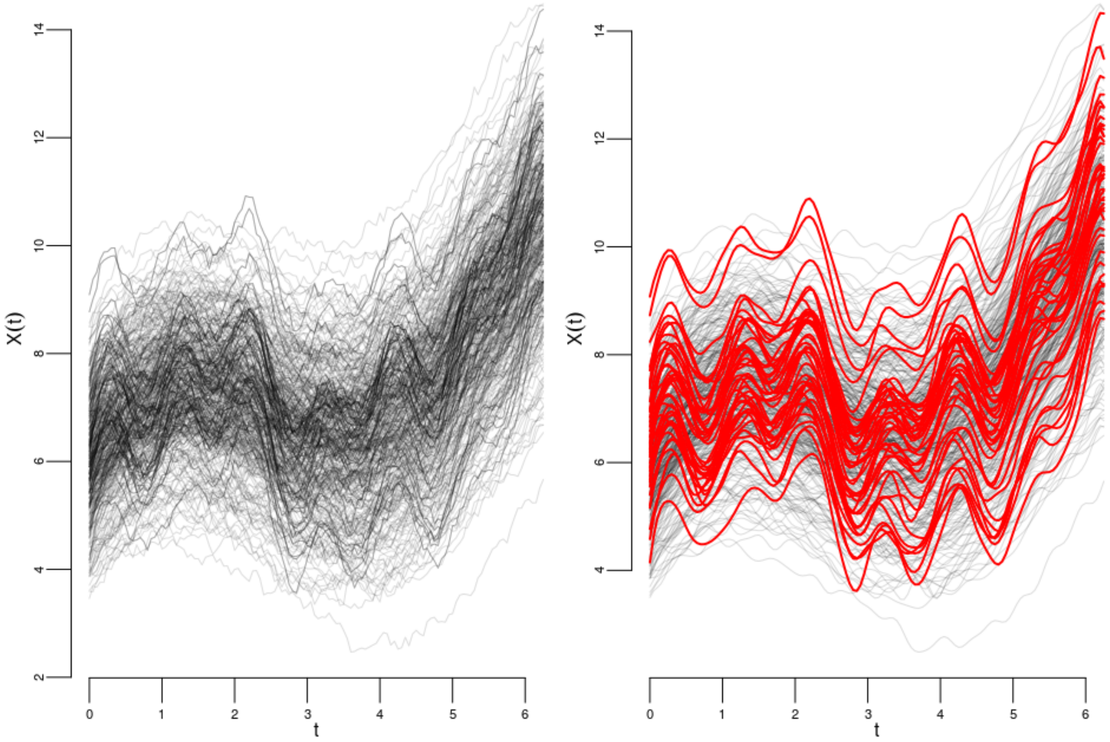

Figure 4. Raw data on the left and functional data on the right. The curves in black (—) are the realization of $X(t)$ and paths in red (—) are the realizations of $Y(t)$.

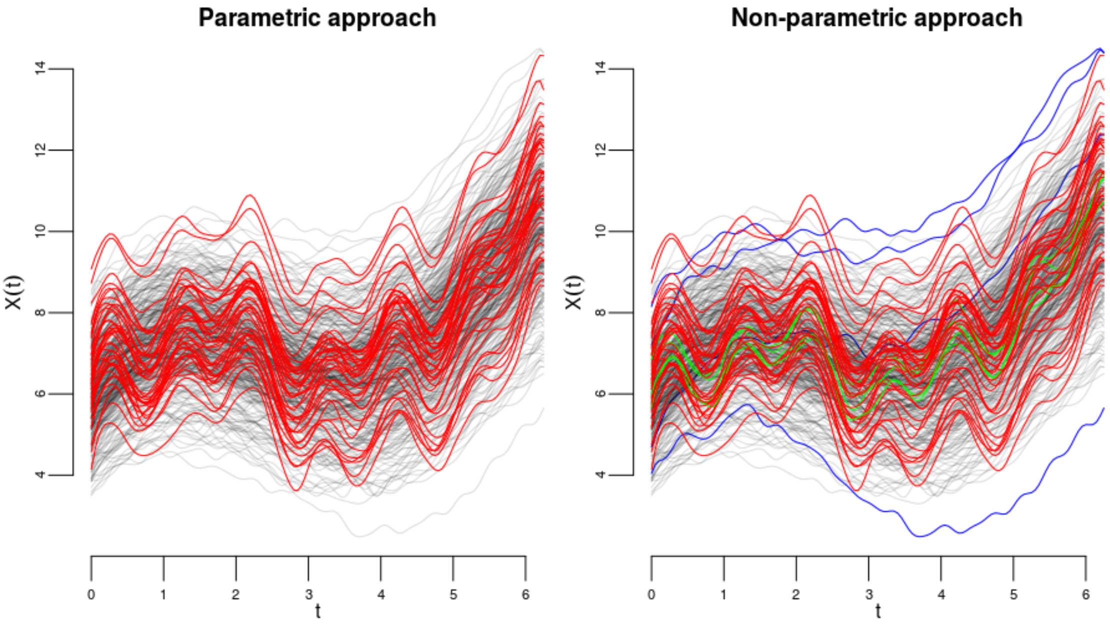

Figure 5. Experimental data: in black (—), normal paths corresponding to the realizations of $X(t)$, in red (—), true outlier detected corresponding to the realizations of $Y(t)$ in blue (—) and false negative in green (—).

corresponds to a value of $\nu = 10\%$ in both cases.

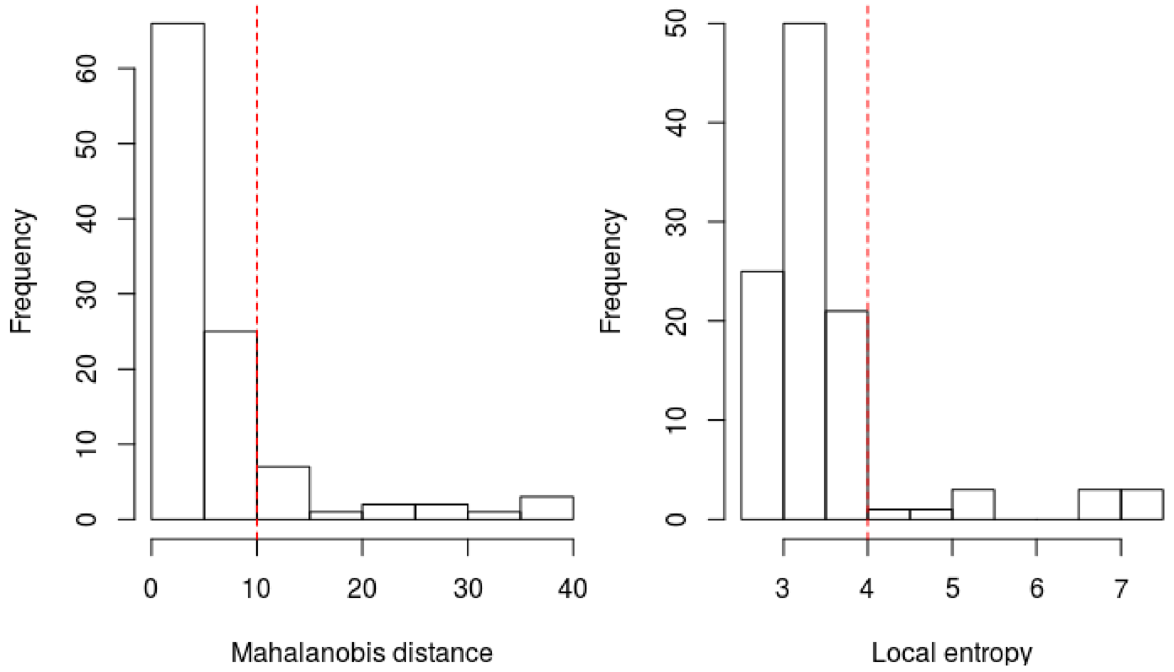

Figure 6. Distribution of the estimated robust Mahalanobis distances (left) and local entropies (right) for the mortality rate dataset. The vertical red line (— — —) denotes the 'elbow' in the distribution of Mahalanobis distance and local entropies, respectively, and corresponds to $\nu = 10\%$ in both cases.

When $\nu = 10\%$, most of the competitor measures identify as anomalous curves the years that correspond to the First World War and the last years of the sample. Only the HMD is able to partially identify as outliers some years corresponding to the Second World War. Even though it is true that for the early 2000s, the mortality rates are the lowest ones, they present the same dynamic as the rest of the years of the sample, so they could be considered as false-positive identifications. The temporal dynamic implicit in the data shows that the mortality rate decreases systematically every year for all the cohorts. This means that a curve that is far from the "center" of the distribution is not necessarily an anomalous curve, but follows the natural dynamics of the process that generates the samples every year.

With respect to the proposed entropy methods, these are able to identify as anomalous curves those years corresponding to the First and Second World War, except for the year 1941. Additionally, the entropy methods are the only ones capable of identifying the year 1919 (influenza pandemic) as an outlying curve. Last, but not least, it is important to mention that for the NPA, the obtained results are robust with respect to the number of neighbors k considered in the method.

Table 2. Anomalous years detected by the different methods for different values of ν.

Metric	Anomalous years					
	$\nu = 50\%$	$\nu = 25\%$	$\nu = 15\%$	$\nu = 10\%$	$\nu = 5\%$	$\nu = 1\%$
MBD	1900–1919; 1922; 1925–1926; 1929; 1940–1944; 1982–2006	1900–1901; 1905–1907; 1909; 1911; 1914–1918; 1940; 1944; 1994–2006	1900; 1907; 1914–1915; 1917–1918; 1940; 1998–2006	1900; 1915; 1918; 1940; 2000–2006	1900; 2002–2006	2004;2006
HMD	1900–1907; 1914–1919; 1934–1954; 1956; 1989–2006	1900; 1914–1919; 1939–1940; 1943–1951; 1998–2006	1914–1919; 1940; 1943–1944; 1946–1948; 2003–2006	1914–1918; 1940; 1943; 1944; 1946; 1947; 2006	1914–1918; 1940; 1944	1918; 1940
RTD	1900–1921; 1925; 1929; 1940; 1943–1945; 1981–2006	1900–1907; 1911; 1914;1919; 1940; 1944; 1996–2006	1900–1901; 1914–1919; 1944; 1998; 2000; 2002–2006	1900; 1914–1918; 2002–2006	1915; 1918; 2003–2006	2005;2006
FSD	1900–1921; 1925–1926; 1940; 1943–1945; 1981–2006	1900–1907; 1914–1919; 1940; 1944; 1995–2006	1900; 1914–1918; 1944; 1998–2006	1914–1918; 2002–2006	1914; 1915; 1918; 2004–2006	1915; 2006
Entropy–PA	**1901; 1904; 1906; 1912; 1914–1922; 1925; 1931–1932; 1934; 1940–1951; 1954; 1959; 1969; 1986–2006**	**1914–1919; 1940; 1942–1945; 1991–2006**	**1914–1919; 1925; 1934; 1940–1945; 2004; 2006**	**1914–1919; 1940; 1942–1945**	**1914–1918;1940**	**1914; 1915**
Entropy–NPA	**1900–1902; 1911; 1914–1919; 1925–1926; 1931; 1940–1945; 1949; 1955; 1957; 1958; 1961–1965; 196–1982; 1988–1995; 1999; 2004–2006**	**1901; 1914–1919 1931; 1940–1945; 1958; 1970–1971; 1974–1975; 1977–1979; 1990–1993; 2006**	**1914–1919; 1931; 1940; 1942–1945; 1970; 1975; 1978; 1992**	**1914–1919; 1940; 1942–1945**	**1914–1918;1940**	**1914; 1915**

The neighbors considered for the NPA were 50.

References

1. López-Pintado S., Romo J. On the concept of depth for functional data. *Journal of the American Statistical Association* 2009: **104**(486), 718–734.
2. Cuevas A., Febrero M., Fraiman R. Robust estimation and classification for functional data via projection-based depth notions. *Computational Statistics* 2007: **22**(3), 481–496.
3. Sguera C., Galeano P., Lillo R. Spatial depth-based classification for functional data. *Test* 2014: **23**(4), 725–750.
4. Cuesta-Albertos J. A., Nieto-Reyes A. The random Tukey depth. *Computational Statistics & Data Analysis* 2008: **52**(11), 4979–4988.

© 2018 by the authors. Submitted to *Entropy* for possible open access publication under the terms and conditions of the Creative Commons Attribution (CC BY) license (http://creativecommons.org/licenses/by/4.0/).
